# Attitudes towards addressing alcohol consumption by healthcare professionals: a cross-sectional study among older adults in the Netherlands

**DOI:** 10.1093/alcalc/agag031

**Published:** 2026-05-18

**Authors:** Wendy Wagenaar, Andrea D Rozema, Jurre M van der Mast, Rob H L M Bovens, Jolanda J P Mathijssen

**Affiliations:** Tranzo, Scientific Center for Care and Wellbeing, Tilburg School of Social and Behavioral Sciences, Tilburg University, Tilburg, Cobbenhagenlaan 125, Tilburg 5037 DB, the Netherlands; Tranzo, Scientific Center for Care and Wellbeing, Tilburg School of Social and Behavioral Sciences, Tilburg University, Tilburg, Cobbenhagenlaan 125, Tilburg 5037 DB, the Netherlands; Tranzo, Scientific Center for Care and Wellbeing, Tilburg School of Social and Behavioral Sciences, Tilburg University, Tilburg, Cobbenhagenlaan 125, Tilburg 5037 DB, the Netherlands; Tranzo, Scientific Center for Care and Wellbeing, Tilburg School of Social and Behavioral Sciences, Tilburg University, Tilburg, Cobbenhagenlaan 125, Tilburg 5037 DB, the Netherlands; Tranzo, Scientific Center for Care and Wellbeing, Tilburg School of Social and Behavioral Sciences, Tilburg University, Tilburg, Cobbenhagenlaan 125, Tilburg 5037 DB, the Netherlands

**Keywords:** older adults, alcohol consumption, healthcare professionals, attitudes, prevention

## Abstract

**Aims:**

Hazardous alcohol consumption represents a substantial health risk for older adults (aged 55 and above). Although healthcare professionals play a key role in addressing alcohol consumption, findings are mixed regarding older adults’ attitudes toward alcohol-related conversations with different professionals. This study examined older adults’ attitudes towards alcohol-related conversations with GPs, practice nurses, district nurses and volunteers, and explored associations with socio-demographic characteristics and alcohol consumption.

**Methods:**

A cross-sectional web-based survey (*N* = 424) was conducted among older adults in the Netherlands, recruited via a national association. Alcohol consumption was assessed using the AUDIT-C and participants were classified as abstainers, low-risk or high-risk drinkers. Attitudes were assessed using six statements. Multivariable logistic regression examined associations between socio-demographic characteristics, alcohol consumption and attitudes.

**Results:**

Support for routinely asking about alcohol consumption was highest for GPs (60.4%, 95% CI 55.7–65.1), followed by practice nurses (55.0%, 95% CI 50.2–59.7), district nurses (46.7%, 95% CI 41.9–51.5) and volunteers (17.5%, 95% CI 13.8–21.1). Older adults aged 75 and above were more likely to view alcohol consumption as a personal matter and less likely to discuss with GPs (OR 0.66, 95% CI 0.43–1.00) or district nurses (OR 0.60, 95% CI 0.38–0.94) than those younger than 75. High-risk drinkers were less supportive of district nurses routinely addressing alcohol consumption (OR 0.54, 95% CI 0.29–0.99) than abstainers and low-risk drinkers.

**Conclusions:**

Attitudes towards alcohol-related conversations differ across professionals, with GPs viewed most favourably and volunteers least. This highlights the need to avoid treating professionals as interchangeable when addressing alcohol consumption.

## Teaser

**Short Summary** Older adults (aged 55 years and above) are at increased risk of alcohol-related harm; however, there are mixed findings regarding their attitudes towards alcohol conversations with healthcare professionals. This study shows that attitudes of older adults vary per healthcare professional: general practitioners are viewed most favourably for alcohol-related conversations, while volunteers receive the least support.

## Introduction

Hazardous alcohol consumption (i.e. drinking patterns that increase health risks without meeting criteria for alcohol use disorder) represents a substantial threat to public health, being a leading global risk factor for morbidity and premature mortality (Griswold et al. 2018, Sohi et al. 2021). Older adults (aged 55 years and above) are particularly vulnerable to the negative effects of alcohol due to physiological changes associated with ageing (Cheitlin 2003, Nigam et al. 2012), a higher prevalence of chronic conditions (Divo et al. 2014, Guo et al. 2022) and increased medication use (Moore et al. 2007). Therefore, alcohol use can increase older adults’ risk of harmful alcohol-related outcomes (Lee et al. 2022). Despite these risks, alcohol consumption among older adults has increased in recent years, with binge drinking (i.e. consuming a large number of drinks on a single occasion) rising from 12.5% to 14.9% and an increase of alcohol use disorders from 3.0% to 3.7% (Kim et al. 2012, Geels et al. 2013, Han et al. 2017, Stelander et al. 2021). In the Netherlands, this causes an increasing demand for addiction care, with the proportion of adults aged 55 years and above seeking treatment for alcohol-related problems rising from 18.0% in 2002 to 28.2% in 2015 (Wisselink et al. 2016).

This growing trend highlights the need for effective interventions targeting hazardous alcohol use. Healthcare professionals, particularly general practitioners (GPs) and nurses, might play a crucial role in addressing alcohol consumption and raising awareness of its potential health effects with their patients (Pedersen et al. 2012, O'Donnell et al. 2014). Standard approaches to address alcohol use include screening for hazardous drinking, providing information on alcohol-related health risks, and offering guidance to reduce alcohol consumption (Bush et al. 1998, Sorocco and Ferrell 2006). Several evidence-based interventions exist, including screening, brief intervention and referral to treatment (SBIRT), cognitive-behavioural therapy, motivational interviewing (Agerwala and McCance-Katz 2012, Cooper 2012, Kaner et al. 2018) and various eHealth interventions to reduce alcohol consumption (Blankers et al. 2011, Riper et al. 2018).

However, despite strong evidence supporting these interventions, healthcare professionals often face challenges in addressing alcohol consumption in practice (Aira et al. 2003, Moriarty et al. 2012, Brown et al. 2015, Kastaun et al. 2022). Studies indicate that various barriers limit the implementation of alcohol screening interventions (Johansson et al. 2005, Johnson et al. 2011). These include organisational challenges (e.g. lack of financial incentives), staff-related barriers (e.g. staff shortage, time constraints) and individual barriers (e.g. limited knowledge and training), but GPs also raise concerns about potential negative reactions of patients (Johnson et al. 2011). Nurses have reported similar concerns, fearing that addressing alcohol consumption, a topic often perceived as private or sensitive, might harm the patient-provider relationship (Johansson et al. 2005).

Despite healthcare professionals’ concerns, research suggests that patients may have a more positive attitude towards alcohol-related conversations than healthcare professionals anticipate (Nilsen et al. 2012). For example, a study in Sweden found that only 1.8% of participants completely agreed with the statement that alcohol use is a personal matter that healthcare professionals should not discuss (Nilsen et al. 2012). Similarly, a study in England reported an agreement rate to this statement of only 10.5% (Nilsen et al. 2012, O’Donnell et al. 2018). Moreover, the study in Sweden demonstrated that older age was associated with an even greater support for healthcare professionals addressing alcohol use (Nilsen et al. 2012). However, not all studies show consistent results. A study conducted in England, the Netherlands, Norway and Sweden found that older adults in the Netherlands held more negative attitudes towards addressing alcohol use in healthcare settings compared to younger age groups (Karlsson et al. 2023). In addition, a Swedish study found that older adults were less likely than younger participants to report a positive effect from alcohol-related conversations with healthcare professionals, such as making them consider their drinking, increasing their understanding of alcohol’s health risks, or leading to a reduction in their drinking (Abidi et al. 2020).

These mixed findings suggest a complex relationship between older adults and alcohol-related conversations in healthcare settings. One potential explanation for these mixed findings is that alcohol conversations can be conducted by different healthcare professionals (Wamsley et al. 2018). These various healthcare professionals may approach alcohol conversations differently, and older adults may have varying perceptions of these interactions depending on the professional involved. In the Netherlands, there are four core professionals important for older adults, which are GPs, practice nurses and district nurses and volunteers (ranging from a formal medical authority towards informal contact with a ‘buddy’) (Verlee et al. 2017). Practice nurses usually work alongside GPs in general practices, while district nurses are based in the community, regularly visiting clients at home and collaborating with professionals from the social care sector (V&VN 2025). Besides these three core professionals, volunteers, often acting as ‘buddies,’ play an increasingly important role in community-based health initiatives (Brabant-Zeeland 2025; Gaber et al. 2020). Volunteers provide informal guidance and practical assistance to older adults, helping them navigate daily challenges. For example, they may offer advice on regulations and available services, assist with applications for healthcare or mobility aids, and provide support in other practical matters (KBO-Brabant 2022). Although previous studies have examined older adults’ attitudes towards alcohol-related conversations in healthcare, there are mixed findings on how these attitudes differ across specific professionals.

Besides differences in attitudes towards various healthcare professionals, personal characteristics may also shape older adults’ attitudes towards alcohol-related conversations. For instance, factors such as gender and education level (e.g. male gender and low educational level), have been associated with more negative attitudes towards alcohol conversations in the general population and may also influence these perspectives (Karlsson et al. 2023). Additionally, individuals with higher levels of alcohol consumption tend to be less open to having alcohol-related conversations (Fankhaenel et al. 2018). However, no research to date has specifically explored how these personal characteristics influence older adults’ attitudes towards alcohol-related conversations with different healthcare professionals. Understanding these dynamics is essential for improving how alcohol consumption is addressed and reducing hazardous alcohol consumption among older adults.

This study has therefore two primary aims: (1) to examine differences in older adults’ attitudes towards alcohol-related conversations with professionals (GPs, practice nurses, district nurses and volunteers); and (2) to explore how socio-demographic factors and alcohol consumption are associated with these attitudes.

## Materials and Methods

### Study population and design

The study employed a cross-sectional design to examine the attitudes of older adults towards alcohol-related conversations with professionals ranging from a formal medical authority (GP) towards informal contact with a ‘buddy’ (volunteer). Data were collected between December 18, 2023, and March 18, 2024, via a web-based survey administered through Qualtrics.

The survey was distributed in collaboration with the *Katholieke Bond van Ouderen Brabant* (KBO Brabant, known as *Senioren Brabant-Zeeland* since August 2024). KBO Brabant is the largest association for older adults in the Netherlands with ~ 120,000 members. Approximately one in three adults aged 75 years and older in the region is a member of a local KBO department, and the average age of members is ~ 76 years (ONS 2022). The survey was disseminated in two ways. First, an article about the study, including a QR-code to participate in the questionnaire, was published in the January 2024 issue of ONS Magazine (the Dutch word for ‘our’). ONS Magazine is the organisation’s magazine, distributed ~ 11 times per year to all members, and includes articles on topics relevant to older adults. Second, local KBO Brabant departments were asked to distribute the survey among their members, who could access it via the provided link or QR-code. The information provided to the participants stated that the study focused on older adults’ views on conversations with healthcare professionals about alcohol use. No specific emphasis was placed on hazardous drinking, and it was specified that participation was voluntary and anonymous. The only inclusion criterion was the participant’s age, which needed to be 55 years or older. As all participants were recruited through KBO Brabant, whose membership primarily consists of older adults aged 65 years and above, all participants were aged 65 years and older and thus met this inclusion criterion. No additional inclusion or exclusion criteria were applied. As recruitment occurred through existing communication channels and participation was self-initiated, precise data on the number of members who viewed the invitation were not available. Participation was therefore based on a self-selected sample.

### Measures

The questionnaire consisted of 12 questions, divided into three sections: (i) socio-demographic characteristics, (ii) alcohol consumption, and (iii) attitudes towards alcohol-related conversations with healthcare professionals.

The first three questions collected socio-demographic data, including age, gender and level of education. A participant’s educational level was measured using predefined response categories and subsequently categorized into three groups: low (primary education and lower secondary education), secondary (upper secondary education and pre-university education), and high (higher professional education or university education). Alcohol consumption was assessed using the Alcohol Use Disorders Identification Test-Consumption (AUDIT-C) (Bush et al. 1998, Babor et al. 2001). The AUDIT-C consists of three questions (Trimbos 2022): (i) *How often have you consumed alcohol in the past 12 months* (never; once a month or less; two to four times per month; two to three times per week; four or more times per week); (ii) *How many standard glasses do you usually drink when consuming alcohol?* (1 or 2; 3 or 4; 5 or 6; 7, 8 or 9; 10 or more); (iii) *How often do you drink six or more glasses on one occasion?* (never; less often than once a month; once a month; once a week; daily or almost daily). Participants received a total AUDIT-C score ranging from 0 to 12, with higher scores indicating more high-risk alcohol use. Based on their responses, participants were categorized into three groups: abstainers, low-risk drinkers and high-risk drinkers. Participants who reported that they had not been drinking any alcohol in the past 12 months were categorized as abstainers. Those with scores below the risk threshold were defined as low-risk drinkers (< 3 for females and < 4 for males based on Wood et al. 2024), indicating a lower risk of alcohol-related problems. Participants scoring at or above the threshold (≥ 3 for females and ≥ 4 for males) were defined as high-risk drinkers, indicating an elevated risk of alcohol-related problems.

Attitudes towards being asked about alcohol use by different healthcare professionals were assessed using six statements in which participants could indicate their answers for every healthcare professional (e.g. GPs, practice nurses, district nurses and volunteers). The first four statements were adapted from previous research examining attitudes towards conversations on alcohol consumption in healthcare settings (O’Donnell et al. 2018, Karlsson et al. 2023). Two additional statements were developed for the present study to capture concerns about potential harm to the patient-professional relationship and willingness to bring up alcohol use themselves. The statements were subsequently pilot tested among a small group of older adults to assess clarity and comprehensibility, leading to minor wording refinements. Hence, the statements include: (i) ‘The healthcare professional below should routinely ask about patients’ alcohol consumption’; (ii) ‘The healthcare professional below should ask about patients’ alcohol consumption, but only if patients seek healthcare to discuss symptoms that could be related to high consumption’; (iii) ‘The healthcare professional below should ask about patients’ alcohol consumption, but only if the issue is brought up by the patient’; (iv) ‘Alcohol consumption is a personal matter and not something the healthcare professional below should ask about’; (v) ‘If the healthcare professional below were to ask about my alcohol use, it would harm our relationship’; (vi) ‘With the healthcare professional below, I would bring up my alcohol use myself’. Participants rated the six statements on a 5-point Likert scale, ranging from ‘strongly disagree’ to ‘strongly agree.’

### Analyses

Statistical analyses were conducted using SPSS 24. Analyses were conducted using complete-case analysis, with participants excluded from a given analysis if data on the relevant variables were missing. Socio-demographic characteristics were analysed using descriptive statistics. Gender, age and education level were compared across drinking categories (abstainers, low-risk drinkers and high-risk drinkers) using chi-square tests. For statistically significant results, pairwise comparisons with Bonferroni correction tests were performed. For further analyses, responses were dichotomized into ‘agreement’ (‘strongly agree’ and ‘agree’) versus ‘non-agreement’ (‘neutral,’ ‘disagree’, and ‘strongly disagree’) to facilitate comparison across healthcare professional groups and ensure statistical power. This approach has been used in previous studies examining attitudes in healthcare professionals (O’Donnell et al. 2018, Karlsson et al. 2023), although the exact categorization may differ. Pairwise comparisons between different healthcare professionals were conducted using McNemar’s test, as each participant provided responses for multiple healthcare professionals, resulting in paired observations. Multivariable logistic regression models were used to examine associations between gender, age, education level (categorical variable), alcohol consumption (categorical variable) and the attitudes of older adults, with all variables entered simultaneously into the models. Results were considered statistically significant at *P* < .05.

## Results

### Sample characteristics

Of the 463 respondents, a total of 424 participants (91.6%) completed the survey and were included in the complete-case analyses (i.e. 8.4% of cases were excluded due to missing data, primarily on items assessing attitudes toward different healthcare providers). Table 1 presents the sociodemographic characteristics of the sample. Among respondents, 13.7% (95% CI 10.4–17.0) were abstainers (N = 58), 38.4% (95% CI 33.8–43.1) were low-risk drinkers indicating a lower risk of alcohol-related problems (N = 163) (AUDIT-C score < 3 for females, < 4 for males) and 47.9% (95% CI 43.1–52.7) were high-risk drinkers, indicating an elevated risk of alcohol-related problems (N = 203) (AUDIT-C score ≥ 3 for females and ≥ 4 for males). There was a statistically significant relationship between alcohol consumption and both gender (χ2(2) = 16.86, *P* < .001) and age (χ2(2) = 6.47, *P* = .039). As is shown in Table 1, high-risk drinkers were significantly more likely to be male and aged 75 years or older. No significant differences in alcohol consumption were found in relation to educational level (χ^2^(4) = 6.12, *P* = .190).

**Table 1:** Sociodemographic characteristics.

Variables	Total, n (%)	Abstainers, n(%, 95% CI)	Low-risk drinkers, n(%, 95% CI)	High-risk drinkers, n(%, 95% CI)
Total	424	58 (13.7, 10.4–17.0)	163 (38.4, 33.8–43.1)	203 (47.9, 43.1–52.7)
**Gender**				
Men	239 (56.4)	19 (7.9, 4.5–11.4)	104 (43.5, 37.2–49.9)	116 (48.5, 42.1–54.9)
Women	185 (43.6)	39 (21.1, 15.2–27.0)	59 (31.9, 25.1–38.7)	87 (47.0, 39.8–54.3)
**Age**				
65–74 years	232 (54.7)	39 (16.8, 11.9–21.7)	93 (40.1, 33.7–46.4)	100 (43.1, 36.7–49.5)
75 years and above	192 (45.3)	19 (9.9, 5.6–14.2)	70 (36.5, 29.6–43.3)	103 (53.6, 46.5–60.8)
**Educational level**				
Low	187 (44.1)	29 (15.5, 10.3–20.7)	77 (41.2, 34.1–48.3)	81 (43.3, 36.2–50.5)
Secondary	193 (45.5)	25 (13.0, 8.2–17.7)	74 (38.3, 31.4–45.3)	94 (48.7, 41.6–55.8)
High	44 (10.4)	4 (9.1, 0.3–17.9)	12 (27.3, 13.6–41.0)	28 (63.6, 48.8–78.4)

*Abbreviations: CI* confidence interval

### Attitudes about addressing alcohol with healthcare professionals

Table 2 presents participants’ attitudes towards addressing alcohol consumption with different healthcare professionals. The results show significant differences in their attitudes regarding these conversations. A majority of 60.4% (95% CI 55.7–65.1) agreed that GPs should routinely ask about alcohol consumption, a significantly higher proportion compared to practice nurses (55.0%, 95% CI 50.2–59.7) and district nurses (46.7%, 95% CI 41.9–51.5). Participants were least supportive of routine alcohol screening by volunteers (17.5%, 95% CI 13.8–21.1).

**Table 2:** Attitudes of participants about addressing alcohol consumption with four healthcare professionals.

Statements					*P* value
	GP n(%, 95% CI)	Practice nurse n(%, 95% CI)	District nurse n(%, 95% CI)	Volunteer n(%, 95% CI)	
**(1) The healthcare professional below should routinely ask about patients’ alcohol consumption**					
Agreement	256 (60.4, 55.7–65.1)	233 (55.0, 50.2–59.7)	198 (46.7, 41.9–51.5)	74 (17.5, 13.8–21.1)	<.001
Non-agreement	168 (39.6, 34.9–44.3)	191 (45.0, 40.3–49.8)	226 (53.3, 48.5–58.1)	350 (82.5, 78.9–86.2)	
**(2) The healthcare professional below should ask about patients’ alcohol consumption, but only if patients seek healthcare to discuss symptoms that could be related to high consumption**					
Agreement	326 (76.9, 72.9–80.9)	287 (67.7, 63.2–72.2)	252 (59.4, 54.7–64.1)	150 (35.4, 30.8–39.9)	<.001
Non-agreement	98 (23.1, 19.1–27.1)	137 (32.3, 27.8–36.8)	172 (40.6, 35.9–45.3)	274 (64.6, 60.1–69.2)	
**(3) The healthcare professional below should ask about patients’ alcohol consumption, but only if the issue is brought up by the patient**					
Agreement	239 (56.4, 51.6–61.1)	213 (50.2, 45.5–55.0)	204 (48.1, 43.3–52.9)	174 (41.0, 36.3–45.7)	<.001
Non-agreement	185 (43.6, 38.9–48.4)	211 (49.8, 45.0–54.5)	220 (51.9, 47.1–56.7)	250 (59.0, 54.3–63.7)	
**(4) Alcohol consumption is a personal matter and not something the healthcare professional below should ask about**					
Agreement	39 (9.2, 6.4–12.0)	58 (13.7, 10.4–17.0)	80 (18.9, 15.1–22.6)	141 (33.3, 28.8–37.8)	<.001
Non-agreement	385 (90.8, 88.0–93.6)	366 (86.3, 83.0–89.6)	344 (81.1, 77.4–84.9)	283 (66.7, 62.2–71.2)	
**(5) If the healthcare professional below were to ask about my alcohol use, it would harm our relationship.**					
Agreement	13 (3.1, 1.4–4.7)	30 (7.1, 4.6–9.5)	47 (11.1, 8.1–14.1)	119 (28.1, 23.8–32.4)	<.001
Non-agreement	411 (96.9, 95.3–98.6)	394 (92.9, 90.5–95.4)	377 (88.9, 85.9–91.9)	305 (71.9, 67.6–76.2)	
**(6) With the healthcare professional below, I would bring up my alcohol use myself.**					
Agreement	274 (64.6, 60.1–69.2)	192 (45.3, 40.5–50.0)	124 (29.2, 24.9–33.6)	44 (10.4, 7.5–13.3)	<.001
Non-agreement	150 (35.4, 30.8–39.9)	232 (54.7, 50.0–59.5)	300 (70.8, 66.4–75.1)	380 (89.6, 86.7–92.5)	

*Abbreviations: CI* confidence interval

Even more participants agreed that alcohol consumption should only be addressed when patients seek healthcare for symptoms that could be related to high alcohol consumption, with the highest level of agreement for GPs (76.9%, 95% CI 72.9–80.9) compared to practice nurses (67.7%, 95% CI 63.2–72.2), district nurses (59.4%, 95% CI 54.7–64.1) and volunteers (35.4%, 95% CI 30.8–39.9). When alcohol use was only addressed if the patient brought it up, participants were more open to volunteers addressing the topic (41.0%, 95% CI 36.3–45.7). However, volunteers remained the least preferred professionals for these conversations compared to GPs (56.4%, 95% CI 51.6–61.1), practice nurses (50.2%, 95% CI 45.5–55.0), and district nurses (48.1%, 95% CI 43.3–52.9).

Only a small proportion (9.2%, 95% CI 6.4–12.0) of respondents believed that alcohol consumption was a personal matter that should not be addressed by GPs, a significantly lower proportion than for practice nurses (13.7%, 95% CI 10.4–17.0), district nurses (18.9%, 95% CI 15.1–22.6) and volunteers (33.3%, 95% CI 28.8–37.8). Concerns about potential harm to the patient-provider relationship were most pronounced with volunteers (28.1%, 95% CI 23.8–32.4), compared to GPs (3.1%, 95% CI 1.4–4.7), practice nurses (7.1%, 95% CI 4.6–9.5), and district nurses (11.1%, 95% CI 8.1–14.1). A significant difference was also observed in participants’ willingness to bring up the topic themselves; 64.6% (95% CI 60.1–69.2) of the participants were most likely to initiate conversations with their GP. In comparison, they were more hesitant to bring it up with practice nurses (45.3%, 95% CI 40.5–50.0), district nurses (29.2%, 95% CI 24.9–33.6), and particularly volunteers (10.4%, 95% CI 7.5–13.3).

### Association between participants’ characteristics and ‘routinely ask about patients’ alcohol consumption’

To better understand the relationship between participant characteristics and older adults’ attitudes towards addressing alcohol consumption, logistic regression analyses were conducted for all six attitude statements. Regression results for four key attitudes (statements 1, 4, 5, and 6) related to the main research aims are presented in the main text, while regression results for the additional exploratory attitudes (statements 2 and 3) are reported in Appendix A. Table 3 presents the logistic regression results for the first outcome ‘routinely ask about patients’ alcohol consumption’. Compared to abstainers, high-risk drinkers were significantly less likely to agree that district nurses should routinely ask about alcohol consumption (OR 0.54, 95% CI 0.29–0.99). No significant associations were found between gender, age or education level and agreeing on routinely asking about patients’ alcohol consumption.

**Table 3:** Logistic regression analyses of ‘routinely ask about patients’ alcohol consumption’.

Variables		GP			Practice nurse		District nurse		Volunteer	
	N	OR^a^	95% CI		OR^a^	95% CI	OR^a^	95% CI	OR^a^	95% CI
Gender										
Men	239	1			1		1		1	
Women	185	1.07	0.71–1.63		1.21	0.80–1.82	1.37	0.91–2.06	0.68	0.40–1.17
Age										
65–74 years	232	1			1		1		1	
75 years or older	192	0.71	0.48–1.07		0.72	0.48–1.08	0.72	0.48–1.08	0.98	0.58–1.66
Education level										
Low	187	1			1		1		1	
Secondary	193	1.25	0.83–1.89		0.83	0.55–1.26	1.07	0.71–1.62	0.79	0.46–1.35
High	44	1.37	0.69–2.72		0.61	0.31–1.19	0.78	0.39–1.54	0.79	0.32–1.94
Alcohol consumption										
Abstainers	58	1			1		1		1	
Low-risk drinkers	163	1.10	0.59–2.08		0.94	0.50–1.76	0.83	0.45–1.55	0.59	0.27–1.26
High-risk drinkers	203	0.87	0.47–1.61		0.83	0.45–1.52	0.54^*^	0.29-0.99	0.67	0.32–1.40

*Abbreviations: OR* odds ratio; *CI* confidence interval

^a^ORs are adjusted for gender, age, education level and alcohol consumption

^*^=significant at *P* < .05

### Association between participants characteristics and ‘alcohol consumption is a personal matter’

Table 4 shows the logistic regression results for ‘alcohol consumption is a personal matter’. Age was significantly associated with this attitude across all four healthcare professionals. Participants aged 75 years or older were more likely to agree that alcohol consumption is a personal matter that should not be addressed by GPs (OR 2.78, 95% CI 1.34–5.68), practice nurses (OR 2.17, 95% CI 1.20–3.91), district nurses (OR 2.06, 95% CI 1.23–3.46) and volunteers (OR 1.75, 95% CI 1.14–2.67). No significant associations were found for gender, education level or alcohol consumption.

**Table 4:** Logistic regression analyses of ‘alcohol consumption is a personal matter’.

Variables		GP			Practice nurse		District nurse		Volunteer	
	N	OR^a^	95% CI		OR^a^	95% CI	OR^a^	95% CI	OR^a^	95% CI
Gender										
Men	239	1			1		1		1	
Women	185	0.81	0.38–1.69		1.16	0.64–2.11	1.47	0.87–2.49	1.20	0.78–1.85
Age										
65–74 years	232	1			1		1		1	
75 years or older	192	2.78^*^	1.34–5.68		2.17^*^	1.20-3.91	2.06^*^	1.23-3.46	1.75^*^	1.14-2.67
Education level										
Low	187	1			1		1		1	
Secondary	193	0.52	0.26–1.05		0.75	0.41–1.35	0.85	0.51–1.43	1.04	0.68–1.61
High	44	-^1^	-		0.68	0.24-1.91	0.69	0.28–1.71	0.90	0.44–1.84
Alcohol consumption										
Abstainers	58	1			1		1		1	
Low-risk drinkers	163	0.75	0.26–2.16		1.01	0.41–2.47	0.69	0.32–1.49	0.79	0.41–1.52
High-risk drinkers	203	0.77	0.28–2.17		0.90	0.38–2.18	0.88	0.42–1.82	1.02	0.54–1.91

*Abbreviations: OR* odds ratio; *CI* confidence interval

^1^Not estimable due to small subgroup size.

^a^ORs are adjusted for gender, age, education level and alcohol consumption

^*^=significant *P* < .05

### Association between participants characteristics and ‘harming the relationship with the professional’

Table 5 presents the results regarding concerns that addressing alcohol consumption may harm the patient-healthcare professional relationship. Participants aged 75 years or older were significantly more likely to believe that addressing alcohol consumption with a district nurse would harm the patient-provider relationship (OR 2.11, 95% CI 1.10–4.03). Education level was also associated with expected potential harm to the relationship with volunteers. Compared to those with a low education level, participants with a secondary education level (OR 1.86, 95% CI 1.17–2.97) or a higher education level (OR 2.38, 95% CI 1.17–4.84) were more likely to agree that asking about alcohol consumption would harm their relationship with the volunteer. Gender and alcohol consumption were not statistically significantly associated with believing that addressing alcohol consumption would harm the relationship.

**Table 5:** Logistic regression analyses of ‘harming the relationship with the professional’.

Variables		GP			Practice nurse		District nurse		Volunteer	
	N	OR^a^	95% CI		OR^a^	95% CI	OR^a^	95% CI	OR^a^	95% CI
Gender										
Men	239	1			1		1		1	
Women	185	0.65	0.19–2.26		0.87	0.39–1.94	1.13	0.59–2.18	1.28	0.82–2.02
Age										
65–74 years	232	1			1		1		1	
75 years or older	192	1.52	0.48–4.81		1.22	0.56–2.64	2.11*	1.10-4.03	1.02	0.65–1.60
Education										
Low	187	1			1		1		1	
Secondary	193	0.60	0.19–1.88		1.22	0.55–2.70	1.21	0.62–2.34	1.86*	1.17-2.97
High	44	-^b^	-		1.11	0.30-4.14	1.33	0.48–3.64	2.38*	1.17-4.84
Alcohol consumption										
Abstainers	58	1			1		1		1	
Low-risk drinkers	163	0.35	0.08–1.49		0.79	0.26–2.40	0.77	0.28–2.17	1.16	0.57–2.33
High-risk drinkers	203	0.25	0.06–1.09		0.67	0.22–2.01	1.16	0.44–3.05	1.15	0.58–2.26

*Abbreviations: OR* odds ratio; *CI* confidence interval

^a^ORs are adjusted for gender, age, education level and alcohol consumption

^b^Not estimable due to small subgroup size.

^c^=significant at *P* < .05

### Association between participants characteristics and ‘bringing up alcohol use myself’

Table 6 presents findings on participants’ willingness to initiate conversations about alcohol use with healthcare professionals. Age was significantly associated with this outcome, as participants aged 75 years or older were less likely to report that they would bring up alcohol use themselves with a GP (OR 0.66, 95% CI 0.43–1.00) or a district nurse (OR 0.60, 95% CI 0.38–0.94). Additionally, participants with a secondary education level were less likely to indicate that they would bring up alcohol use with a practice nurse (OR 0.62, 95% CI 0.41–0.93). No significant associations were found for gender or alcohol consumption.

**Table 6:** Logistic regression analyses of ‘bringing up alcohol use myself’.

Variables		GP			Practice nurse		District nurse		Volunteer	
	N	OR^a^	95% CI		OR^a^	95% CI	OR^a^	95% CI	OR^a^	95% CI
Gender										
Men	239	1			1		1		1	
Women	185	0.93	0.61–1.43		1.00	0.66–1.51	1.36	0.87–2.12	0.77	0.39–1.53
Age										
65–74 years	232	1			1		1		1	
75 years or older	192	0.66^*^	0.43–1.00		0.67	0.45–1.01	0.60^*^	0.38-0.94	1.57	0.82–3.02
Education										
Low	187	1			1		1		1	
Secondary	193	1.12	0.74–1.72		0.62^*^	0.41-0.93	0.85	0.54–1.33	0.61	0.32–1.18
High	44	1.65	0.79–3.45		0.91	0.47–1.78	1.01	0.49–2.10	0.32	0.07–1.40
Alcohol consumption										
Abstainers	58	1			1		1		1	
Low-risk drinkers	163	1.50	0.79–2.82		1.38	0.74–2.58	1.21	0.63–2.34	0.56	0.23–1.39
High-risk drinkers	203	1.29	0.70–2.38		1.06	0.58–1.95	0.79	0.41–1.51	0.49	0.20–1.20

*Abbreviations: OR* odds ratio; *CI* confidence interval

^a^ORs are adjusted for gender, age, education level and alcohol consumption

^b^=significant *P* < .05

## Discussion

### Key findings

This study examined older adults’ attitudes towards alcohol-related conversations with professionals ranging from a formal medical authority towards informal contact with volunteers, often acting as ‘buddies.’ The findings indicate that attitudes towards alcohol-related conversations vary considerably depending on the professional involved (i.e. GPs, practice nurses, district nurses, and volunteers). Older adults were most supportive of GPs routinely addressing alcohol consumption, whereas support was considerably lower for volunteers. In addition, older adults aged 75 years and above were more likely to view alcohol use as a personal matter and less likely to initiate conversations with GPs or district nurses compared to those younger than 75. High-risk drinkers also showed less support for district nurses routinely addressing alcohol consumption, compared to abstainers and low-risk drinkers.

### Interpretation of the key findings

Previous research has shown mixed findings regarding older adults’ attitudes towards addressing alcohol consumption in healthcare settings (Nilsen et al. 2012, O’Donnell et al. 2018, Abidi et al. 2020, Karlsson et al. 2023). This study suggests that these mixed findings may be partly explained by differences in how older adults perceive various healthcare professionals. Specifically, older adults appear to prefer alcohol-related conversations with more formal medical authorities such as GPs. While practice nurses were viewed slightly less favourably than GPs, they were still viewed positively as healthcare professionals who could routinely address alcohol use. Overall, our results highlight that older adults’ attitudes towards alcohol-related conversations vary considerably depending on the type of professional involved, with greater acceptance of formal medical authorities such as GPs, and notably lower support for more informal contacts such as volunteers.

In addition to differences across healthcare professionals, we found that several personal characteristics were associated with attitudes towards alcohol-related conversations. Specifically, the data show that age was an important factor in different attitudes towards addressing alcohol consumption. Older adults aged 75 years or older were more likely to agree that alcohol consumption is a personal matter that should not be addressed by GPs, practice nurses, district nurses and volunteers. They were also less likely to report willingness to initiate conversations about alcohol themselves with a GP or a district nurse. These findings build on previous research indicating that older individuals (aged 50+) are generally less receptive to alcohol-related conversations in healthcare (Karlsson et al. 2023), but extend this understanding by identifying a stronger effect in the 75+ age group. One potential explanation could lie in generational norms, as older adults may fear being stigmatized as risky drinkers (Wilson et al. 2013) or may not recognize alcohol-related risks as relevant to them (Bareham et al. 2019). Such generational norms are comparable to patterns observed in attitudes towards mental health, where older adults tend to emphasize independence and privacy and are often more reluctant to engage in conversations about sensitive or potentially stigmatizing issues (Teo et al. 2022, Elshaikh et al. 2023). Besides generational norms, this pattern may reflect age-related changes. For example, with increasing age, sensory and cognitive changes (e.g. reduced hearing acuity, slower processing speed) may make complex or sensitive conversations more demanding (Lu et al. 2024, Manchha et al. 2025). This presents a dilemma for healthcare professionals, as this age group is simultaneously more vulnerable to the negative effects of alcohol due to physiological changes associated with ageing and interactions between alcohol use and increased medication use (Cheitlin 2003, Moore et al. 2007, Nigam et al. 2012). Future research should take this nuance into account, especially when designing interventions for older subpopulations, and further explore whether these patterns reflect cohort-related norms, age-related changes, or a combination of both.

Another finding relates to older adults’ alcohol consumption. High-risk drinkers were more likely to hold negative attitudes towards routine alcohol conversations, particularly when initiated by district nurses. This aligns with previous studies suggesting that individuals with higher levels of alcohol consumption tend to be less open or more negative when addressing alcohol consumption (Fankhaenel et al. 2018, Lid et al. 2021). These findings emphasize the need for tailoring alcohol-related conversations not only based on the type of professional but also on older adults’ alcohol consumption. In addition, although our findings show that male gender was significantly associated with higher alcohol consumption patterns overall, the proportion of participants classified as high-risk drinkers was high for both men and women. This underscores the need for inclusive approaches that address older adults of all genders.

Based on previous research, a key concern among healthcare professionals was the potential to harm the patient-provider relationship by asking about alcohol use, causing reluctance to address alcohol consumption (Johansson et al. 2005, Johnson et al. 2011). Our results suggest that concerns about harming the relationship are valid, specifically when volunteers are involved (28.1%). These concerns are less applicable for GPs (3.1%), practice nurses (7.1%) and district nurses (11.1%). Older adults aged 75 years and above were especially likely to worry that such conversations with district nurses could damage the patient-provider relationship. A potential explanation is that this age group is more likely to receive care from district nurses in their own home. Discussing sensitive topics such as alcohol use in the home environment may increase feelings of vulnerability, particularly when a healthcare professional enters a private space. As a result, older adults may perceive conversations about alcohol consumption as more intrusive or inappropriate in this context. In addition, older adults with secondary or higher education were more likely to believe that raising the issue with a volunteer could negatively affect their relationship, compared to older adults with lower education levels. A potential explanation might be that individuals with higher education levels may have stronger expectations regarding professional expertise, making such conversations with volunteers feel more inappropriate. However, more research is needed to further investigate this.

In summary, these findings highlight the need for healthcare systems to avoid treating all healthcare professionals as interchangeable when addressing alcohol use. Although this study identifies clear differences in how various professionals are perceived, it does not fully explain why these differences exist or what specific elements influence these attitudes. This is partly related to the quantitative design of the study, which captures general patterns in attitudes but offers limited insight into the underlying meanings, experiences, and relational factors that shape these perceptions. One potential explanation lies in the variation of roles and perceived authority among these professionals. For instance, GPs and practice nurses are often regarded as more formal sources of health advice due to their medical status, whereas volunteers may be seen as less authoritative. Another important distinction may lie in the personal relationship and established trust. GPs and practice nurses are not only formal medical authorities but are also professionals with whom older adults often have long-standing, personal relationships. This may help explain the high levels of acceptance observed for GPs and practice nurses and the finding that many participants indicated they would themselves initiate alcohol-related discussions with their GP. In addition, gender distributions across these professional roles may also play a role, as some roles (e.g. district nurses) are more often held by women than others. This may be particularly relevant among older adults, as research suggests that they may show a preference for women as professionals involved in interventions to reduce alcohol consumption (Knijff et al. 2025). To further investigate this, qualitative research is needed to explore the underlying beliefs and expectations that shape attitudes towards different healthcare roles, including trust and medical authority. Additionally, as GPs increasingly shift towards addressing lifestyle factors and are moving beyond the earlier focus solely on detecting hazardous alcohol consumption (NHG 2025), qualitative research could also examine how this new lifestyle-oriented approach influences attitudes towards addressing alcohol consumption with GPs and lowers the threshold for having these conversations. Such insights would be valuable in designing more effective interventions that align with patient expectations and preferences.

While this study offers valuable insights, several limitations should be acknowledged. First, several subgroup regression estimates were accompanied by wide confidence intervals, and for some subgroups estimates could not be calculated due to small numbers within specific subgroups, such as highly educated older adults. In particular, for some attitude statements the number of participants expressing agreement was very low, resulting in insufficient events for reliable estimation in subgroup analyses. As wide confidence intervals reflect substantial uncertainty around the estimated effects, the observed subgroup differences should be viewed as indicative rather than conclusive. Second, the sample included a relatively small proportion of highly educated older adults, which may have influenced the findings and limited statistical power in subgroup analyses. A more balanced sample could potentially offer a deeper understanding of how education influences attitudes. However, the proportion of highly educated older adults in our sample reflects national trends; for instance, in 1981, only 11.1% of older adults in the Netherlands had a high educational level, which may also reflect the more limited educational opportunities available to this age group (van der Mooren and de Vries 2022). Additionally, the participants of this study include a high number of low-educated older adults, a group that is often underrepresented in research. Third, the recruitment strategy relied on voluntary participation through an older adults’ association, which may have introduced self-selection bias. Individuals with a greater interest in health or alcohol-related topics may have been more likely to participate. This self-selection may have biased the results, as individuals who were more open to discussing alcohol use may have been more likely to participate, which may partly explain the relatively high proportion of participants classified as high-risk drinkers. However, national data indicate that approximately half of adults aged 50 years and above in the Netherlands report drinking no more than one alcoholic drink per day (CBS 2024). Within the AUDIT-C scoring system, drinking one alcoholic drink per day can already result in a score that meets the threshold for high-risk drinking, suggesting that the observed proportion of higher-risk drinkers in this study may not deviate strongly from patterns observed in the general population. Relatedly, to enable comparisons across professional groups and to maintain sufficient statistical power, response categories were dichotomized in the analyses. While this approach is commonly used in comparable research (O’Donnell et al. 2018, Karlsson et al. 2023), it may have obscured nuance, as neutral responses could reflect a distinct position rather than simple non-agreement. This should be taken into account when interpreting the findings. Finally, although KBO members constitute a substantial proportion of older adults in the Netherlands, they may differ from non-members in terms of social engagement, health awareness, and willingness to participate in research. Despite these limitations, the study is valuable in capturing the perspectives of a diverse group of older adults, including a substantial proportion of lower-educated older adults who are often underrepresented in research. In addition, the study uniquely compares attitudes of older adults towards alcohol-related conversations across different healthcare professionals, ranging from GPs to volunteers. This provides more nuanced insight into how older adults perceive alcohol-related conversations in different care contexts.

## Conclusion

The results of this study show that older adults’ attitudes towards addressing alcohol consumption vary considerably depending on the type of healthcare professional involved, as well as individual characteristics. These findings highlight the need for healthcare systems to avoid treating all professionals as interchangeable when addressing alcohol use. In addition, they underscore the importance of adopting tailored approaches that take individual characteristics, including age and alcohol consumption, into account when engaging older adults in conversations about alcohol consumption. This, in turn, might contribute to healthier ageing by supporting more appropriate prevention and intervention strategies for hazardous alcohol use among older adults.

## Data Availability

The data associated with the current study are available from the corresponding author upon reasonable request.

## Appendix

**Table A1:** Logistic regression analyses of ‘The healthcare professional below should ask about patients’ alcohol consumption, but only if patients seek healthcare to discuss symptoms that could be related to high consumption’.

Variables		GP			Practice nurse		District nurse		Volunteer	
	N	OR^a^	95% CI		OR^a^	95% CI	OR^a^	95% CI	OR^a^	95% CI
Gender										
Men	239	1			1		1		1	
Women	185	1.55	0.96–2.50		1.35	0.87–2.09	1.00	0.66–1.52	0.95	0.62–1.45
Age										
65–74 years	232	1			1		1		1	
75 years or older	192	1.10	0.68–1.79		0.86	0.56–1.33	0.90	0.60–1.36	0.73	0.48–1.12
Education level										
Low	187	1			1		1		1	
Secondary	193	0.59^*^	0.36-0.98		0.49^*^	0.31-0.76	0.60^*^	0.39-0.90	1.10	0.72–1.69
High	44	0.52	0.24–1.10		0.58	0.28–1.17	0.90	0.45–1.79	1.03	0.51–2.08
Alcohol consumption										
Abstainers	58	1			1		1		1	
Low-risk drinkers	163	1.09	0.53–2.23		1.14	0.59–2.23	1.17	0.52–2.21	1.16	0.62–2.20
High-risk drinkers	203	1.10	0.55–2.20		0.92	0.48–1.75	0.75	0.41–1.39	0.90	0.48–1.69

*Abbreviations: OR* odds ratio; *CI* confidence interval

^a^ORs are adjusted for gender, age, education level and alcohol consumption

^*^=significant *P* < .05

**Table A2:** Logistic regression analyses of ‘The healthcare professional below should ask about patients’ alcohol consumption, but only if the issue is brought up by the patient’.

Variables		GP			Practice nurse		District nurse		Volunteer	
	N	OR^a^	95% CI		OR^a^	95% CI	OR^a^	95% CI	OR^a^	95% CI
Gender										
Men	239	1			1		1		1	
Women	185	1.52^*^	1.01-2.30		1.22	0.81–1.84	1.24	0.82–1.86	1.01	0.67–1.54
Age										
65–74 years	232	1			1		1		1	
75 years or older	192	1.11	0.74–1.66		1.02	0.68–1.52	0.89	0.60–1.33	0.84	0.56–1.26
Education										
Low	187	1			1		1		1	
Secondary	193	0.74	0.49–1.11		0.68	0.45–1.02	0.74	0.49–1.11	1.01	0.67–1.52
High	44	0.63	0.32–1.23		0.55	0.28–1.07	0.56	0.29–1.11	0.44^*^	0.21-0.92
Alcohol consumption										
Abstainers	58	1			1		1		1	
Low-risk drinkers	163	0.80	0.43–1.49		0.78	0.42–1.45	0.90	0.49–1.66	1.58	0.83–3.01
High-risk drinkers	203	0.95	0.52–1.75		0.77	0.42–1.41	0.91	0.50–1.66	1.61	0.86–3.03

*Abbreviations: OR* odds ratio; *CI* confidence interval

^a^ORs are adjusted for gender, age, education level and alcohol consumption

^*^=significant *P* < .05
